# Oxidative Stress Induced by Selenium Deficiency Contributes to Inflammation, Apoptosis and Necroptosis in the Lungs of Calves

**DOI:** 10.3390/antiox12040796

**Published:** 2023-03-24

**Authors:** Jing Mu, Lei Lei, Yingce Zheng, Jia Liu, Jie Li, Ding Li, Guanbo Wang, Yun Liu

**Affiliations:** 1 Key Laboratory of Comparative Medicine, Department of Veterinary Surgery, College of Veterinary Medicine, Northeast Agricultural University, Harbin 150030, China; b20060119@neau.edu.cn (J.M.); b20060208@neau.edu.cn (L.L.); s220601088@neau.edu.cn (J.L.); s220602059@neau.edu.cn (D.L.); 2 College of Life Science, Northeast Agricultural University, Harbin 150030, China; zhengyingce@neau.edu.cn; 3 Veterinary Medical Teaching Hospital, Northeast Agricultural University, Harbin 150038, China; liujia@neau.edu.cn; 4 Wellcome-Wolfson Institute for Experimental Medicine, Queen’s University Belfast, Belfast BT7 1NN, UK

**Keywords:** calves, selenium deficiency, lung injury, oxidative stress, pathomechanism

## Abstract

Selenium is an essential trace element for health that can only be obtained through food. However, the pathological processes of selenium deficiency in cattle have received little attention. This study investigated the effects of selenium deficiency on oxidative stress, apoptosis, inflammation, and necroptosis in the lungs of weaning calves compared with healthy calves as controls. The lung selenium content and the expression of 11 selenoproteins mRNA in selenium-deficient calves were substantially reduced compared with the controls. Pathological results showed engorged alveolar capillaries, thickened alveolar septa, and diffuse interstitial inflammation throughout the alveolar septa. The levels of GSH and T-AOC, as well as the CAT, SOD, and TrxR activities, were significantly decreased compared with healthy calves. MDA and H_2_O_2_ were significantly elevated. Meanwhile, the apoptosis activation in the Se-D group was validated. Next, in the Se-D group, several pro-inflammatory cytokines showed higher expression. Further research revealed that the lungs in the Se-D group experienced inflammation via hyperactive NF-κB and MAPK pathways. The high level of expression of *c-FLIP*, *MLKL*, *RIPK1*, and *RIPK3* indicated that necroptosis also causes lung damage during selenium deficiency.

## 1. Introduction

Selenium (Se) is essential for organisms and is always involved in redox reactions. There are a number of diseases that can be caused by a deficiency of Se, including cardiovascular disease, neuromuscular disease, inflammation, and so on. Se is only available through the diet, so the intake of Se has a direct impact on health. Diseases caused by Se deficiency have occurred on occasion because most of China’s soil is deficient in Se [1]. Se deficiency symptoms in humans can be identified and treated quickly, whereas in animals they are frequently ignored [2].

Se functions in organisms through Se-containing proteins, which have been extensively researched [3]. Se is primarily encoded as selenocysteine (Sec) by the codon UGA during translation. UGA codons normally signal translation termination, but the Sec machinery enhances the coding potential of UGA by interacting with the canonical translation machinery. With the assistance of SECIS binding protein 2 (SBP2) and Sec-specific translation elongation factor, UGA was eventually recoded into Sec [3]. Most selenoproteins are enzymes involved in redox reactions, such as glutathione peroxidases (GPX), thyroid hormone deodinases (DIO), and thioredoxin reductases (TXNRD or TrxR). Deletions of Sec result in enzyme inactivation because Sec residues are always in the active site. This is the primary reason why Se deficiency causes redox disorders.

The effects of Se on lung function are primarily in the anti-infective, antioxidant, and alveolar development capacities. It has been demonstrated that macrophage-specific host selenoprotein restricts intracellular bacterial proliferation and protects mice from *Francisella tularensis* infection [4]. The antioxidant GPX protects respiratory membranes from peroxidative damage [5]. Mouse lung fibroblasts with low *GPX4* expression were significantly more susceptible to H_2_O_2_, cadmium, and hydrogen peroxide-induced cytotoxicity [6]. In a model of *Mycobacterium tuberculosis* infection, *GPX4*-deficient mice exhibited more severe lung necrosis and increased bacterial counts. In addition, *GPX4* has been shown to attenuate pulmonary necrosis caused by *Mycobacterium tuberculosis* infection by modulating ferroptosis [7]. The *TXNRD1* is primarily expressed by airway epithelial cells and protects the lungs via an Nrf2-dependent mechanism [8]. Adequate dietary Se raises blood levels of the cytokines IL-6 and IFN-γ, which may reduce susceptibility to disease caused by changes in IFN-γ expression, at least in part [9]. The deficiency of *SEL N* also causes abnormal lung development with enlarged alveoli, which is related to a reduction in tissue elasticity and an increase in quasi-static compliance [10].

Multiple animal models have demonstrated that Se deficiency causes the generation of ROS (reactive oxygen species) that damage cellular lipids, DNA, and proteins [11]. ROS accumulation and oxidation are always mitigated by the antioxidant effects of SOD (superoxide dismutase), CAT (catalase), TXNRD, and GPX. Besides resisting oxidative damage, these enzymes also act as oxidative sensors for the cellular response to ROS. It has been proven that NF-κB (nuclear factor kappa-B) is regulated by intracellular redox status [12]. ROS reduce their own levels by NF-κB to promote cell survival [13]. There is evidence that the NF-κB pathway regulates *TXNRD1* and *TXNRD2*, two of the most important intracellular antioxidants in skeletal muscle cells. TXNRD protects cells from oxidative stress by reacting with ROS via their active site, which results in less oxidized proteins. MAPK (mitogen-activated protein kinase) has important regulatory roles in proliferation, apoptosis, and migration in cells [14]. According to some studies, activation of MAPK and ERK increases antioxidant capacity and prevents cell death. However, it is unclear whether the NF-κB and MAPK pathways are regulated in Se-deficient calves.

Apoptosis is an animal-specific type of cell death that is characterized by a reliance on caspase activity [15]. The initiators *Caspase-8* and *Caspase-9* activate two final executors, *Caspase-3* and *Caspase-7*, ultimately causing apoptosis. In this study, the effect of Se deficiency in calves on apoptosis in lung cells was investigated. Hyperoxia exposure has been reported to produce pulmonary oxidative stress, leading to necrosis and lung injury in rats. *MLKL* (mixed lineage kinase domain-like) is one of the major causes of necroptosis, and it is controlled by *RIPK1* and *RIPK3*. Autophosphorylated *RIPK3* phosphorylates *MLKL* by psKD, leading to conformational changes in the *MLKL* support region followed by pore formation and fracture of the cell [16].

Lungs are important organs for respiration in mammals, and many studies have shown that Se contributes to lung development [17,18]. Se deficiency is associated with worse respiratory health in humans during childhood and infancy, while Se deficiency enhances the susceptibility of neonatal lungs to injury [19]. When the antioxidant capacity of the lungs is reduced, higher oxygen exposure compared with other organs is more likely to cause oxidative stress. In this study, Se-deficient calves showed significant respiratory distress and large amounts of pleural effusion accompanied by pulmonary edema and congestion during the field dissection. Although severe damage to the myocardium and heart failure were found in Se-deficient calves in a previous study [20], it has been suggested that pulmonary edema during Se deficiency may not be entirely due to heart failure [21]. Studies on the effect of Se on lung function have always focused on mice and humans, with no relevant studies in calves. So the present study investigated the mechanism of Se deficiency on lung injury in weaned calves, which is important for mitigating tissue damage caused by Se deficiency.

## 2. Materials and Methods

### 2.1. Experimental Animals

The study was divided into two groups, each consisting of five female weaned calves at about 60 days of age. The animals in the Se-D group were from a farm in Heilongjiang province, where a dozen calves from the same bullpen presented with diarrhea, edema, respiratory distress, and even death within one week of weaning. This study performed a rigorous autopsy and laboratory diagnosis of the affected calves and ruled out bovine viral diarrhea virus (BVDV), infectious bovine rhinotracheitis virus (IBRV), Coccidia, Cryptosporidium, Pasteurella, and other bacterial infections. The Se levels in the blood and tissues of affected calves were significantly lower than in normal individuals, and in combination with the therapeutic diagnosis (weaned calves with mild symptoms recovered after Se supplementation), the final diagnosis of the deficiency disease was confirmed. Five affected individuals were included in the Se-D group, while five healthy weaned calves from another farm of the same breed and age were selected as the Se-C group. All animals were fed and watered ad libitum and had the same food structure, which contained 4/5 of feed and 1/5 of alfalfa. Five portions of each food were randomly collected for Se content examination. Animals were executed after electroshock in accordance with the Animal Care and Use Committee of the Institute of Animal Science, Northeast Agricultural University (SRM-11) restrictions. The lungs were quickly collected and rinsed with saline, then transported to the laboratory at 4 °C. Some tissues were fixed with 4% neutral paraformaldehyde, while others were stored at −80 °C for subsequent experiments.

### 2.2. Detection of Se Concentration in Lung Tissue

The Agilent 7800 ICP-MS (Santa Clara, CA, USA) was used to measure the Se concentrations in lungs in line with the National Standard of China (GB 5009.268).

### 2.3. Pathological Examination and TUNEL Staining

Tissues fixed with formalin were dehydrated and embedded in paraffin wax, followed by cutting into 5 μm slices. The tissue sections were observed under a Nikon Microphot SA microscope (Nikon, Tokyo, Japan) after staining with hematoxylin and eosin. This study stained tissue sections with the TUNEL kit (Vazyme, Nanjing, China). Fluorescence microscopy was used to acquire images, five fields per coverslip (×200 magnification). In order to calculate the apoptotic index, TUNEL-positive cells were counted, and the DAPI stain was used to measure the total number of cells.

### 2.4. Detection of Oxidative Stress Indicators and Cytokines

At cryogenic temperatures, the minced lung was homogenized in saline by an Ultra-Turrax homogenizer (IKA, Staufen, Germany). The supernatant was harvested for the next step, after the homogenate of lung tissue was centrifuged for 15 min at 3000 rpm. Commercial kits (Jiancheng, Nanjing, China) were used to measure redox indicators. All protocols were conducted as described in the manufacturer’s instructions. All of the kits this study used in this section are listed below: GSH (glutathione) concentration, CAT, H_2_O_2_ concentration, TrxR, SOD, MDA (malondialdehyde) concentration, and T-AOC (total antioxidant capacity). ELISA kits from the Jingmei Institute of Biotechnology (Jingmei, Wuxi, China) were used to detect cytokine levels. Microplate readers (BioTek Epoch, Winooski, VT, USA) were used to measure the absorbance. The standard curve R^2^ value was >0.990, the inter-plate and intra-plate variabilities were <15%. The BCA kits (Meilunbio, Dalian, China) were used to assess protein concentrations, which were used to standardize all the data in this section.

### 2.5. RT-qPCR

RNA in lung tissue was extracted by TRIzol (Invitrogen, Carlsbab, CA, USA). The purity of RNA was evaluated with Nano Drop (Thermo, Wilmington, NC, USA). The PrimeScript RT kit (Takara, Dalian, China) was used to synthesize the cDNA in accordance with the manufacturer’s guidelines. The primer sequences were used by referring to the previous research [20]. 2× SYBR Green qPCR Master Mix was used to perform RT-qPCR assays detected with the Roche LightCycler 480II system (Basel, Switzerland). The RT-qPCR was conducted in 20 μL reaction volumes that contained primers (0.4 μM), SYBRs Premix Ex TaqTM reagents (10 μL, Takarabiomed, Tokyo, Japan), cDNA (2 μL, 5-fold dilutions), and ddH_2_O (6.4 μL). *GADPH* was set as a reference for mRNAs.

### 2.6. Western Blotting

Lung tissue was ground at low temperature in RIPA lysis buffer (ThermoFisher, Waltham, MA, USA) containing 1% PMSF (Beyotime Bio, Shanghai, China) with an Ultra-Turrax homogenizer. A BCA Protein Assay Kit (Meilunbio, Dalian, China) was used to determine the protein content of samples. The samples (3 μg/μL) were boiled for 10 min after being mixed with the SDS loading buffer (Sevenbio, Beijing, China). The present study separated the proteins using SDS-PAGE. In the next step, proteins were transferred to nitrocellulose membranes and blocked with 10% BSA for 2 h. Furthermore, the blots were incubated overnight at 4 °C with a primary antibody. The internal reference was either *GAPDH* or *β-actin*, according to the size of the protein. After washing with 1×TBST, a secondary antibody (Abclonal, Wuhan, China) was incubated on the membranes at room temperature. The primary antibodies are listed in Supplementary Table S1. Enhanced chemiluminescence (ECL) plus (Meilunbio, Dalian, China) was used for band visualization. Blots were exposed by the Tanon 5200 imaging system (Bio Tanon, Shanghai, China). Quantification of the Western blot was performed using Image J software.

### 2.7. Statistical Analysis

All analyses for statistical differences were performed using SPSS version 27.0 (SPSS, Chicago, IL, USA). To verify the normal distribution of data, the Shapiro-Wilk test was applied. Two-tailed Student’s *t*-tests and Mann-Whitney U tests were used when comparing the two groups. *p* < 0.05 was considered statistically significant (* 0.01 < *p* < 0.05, ** 0.001 < *p* < 0.01, and *** *p* < 0.001), ns represents no statistically significant. All experiments were performed three times.

## 3. Results

### 3.1. Se Content in Samples and Selenoprotein Expression Levels in Calves’ Lungs

**Figure 1:**
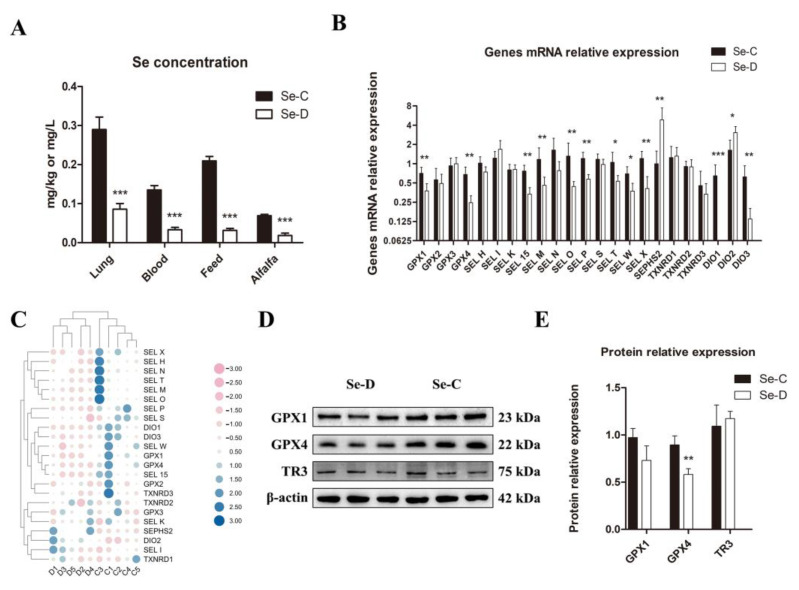
Se contents in lungs, blood, and foods (**A**), selenoprotein expression at gene (**B**,**C**) and protein (**D**,**E**) levels between Se-deficient and healthy calves. The mean ± SD is used to present the data above, *n* = 5 calves per group (except for WB *n* = 3). * *p* < 0.05, ** *p* < 0.01, *** *p* < 0.001.

The study examined the Se content in the lungs, blood, and foods of both groups. The results showed a significantly decreased Se content in all the samples of the Se-D group compared with the controls (Figure 1A). For the reason that the selenoprotein levels reflect the level of Se in the organism to some extent, this study investigated the expression of 23 selenoproteins using RT-qPCR. The results showed that there were 11 selenoproteins with significantly lower gene expression levels in the Se-D group: GPX1, GPX4, SEL W, SEL X, SEL M, SEL T, SEL O, SEL P, SEL 15, DIO1, and DIO3. The gene expression levels of SEPHS2 and DIO2 were significantly higher in the Se deficient calves, whereas there were no significant differences in the other 10 selenoproteins between Se deficient and healthy calves (Figure 1B,C). The protein levels of selenoprotein GPX1, GPX4, and TXNRD3 were examined by WB, and the results showed that Se-deficient calves had significantly lower GPX4 levels than healthy calves. However, there was no significant difference in the expression of the other two selenoproteins (Figure 1D,E).

### 3.2. Pathology of the Lung Tissue in Se-Deficient Calves

**Figure 2:**
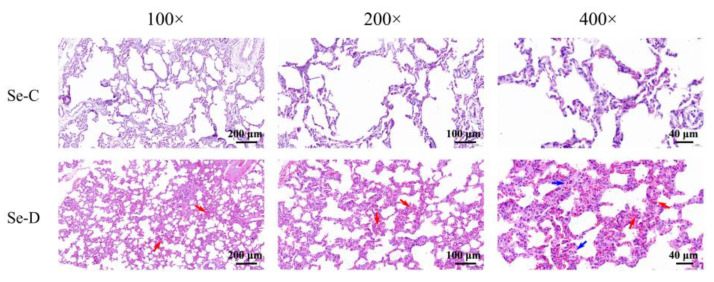
Pathological changes in the lungs of Se-deficient calves (**lower**) compared with healthy calves (**upper**) in pathological histological examination. Red arrows mark pulmonary congestion and blue arrows mark inflammatory infiltrates.

In the control group, the lungs appear well-organized and structurally normal. In contrast, the Se-D group had swollen alveolar epithelial cells and thickened alveolar septa, engorged alveolar capillaries (red arrows), and increased diffuse interstitial inflammation (blue arrows) throughout the alveolar septa (Figure 2).

### 3.3. Determination of Redox Capacity of Lung Tissue in Se-Deficient Calves

**Figure 3:**
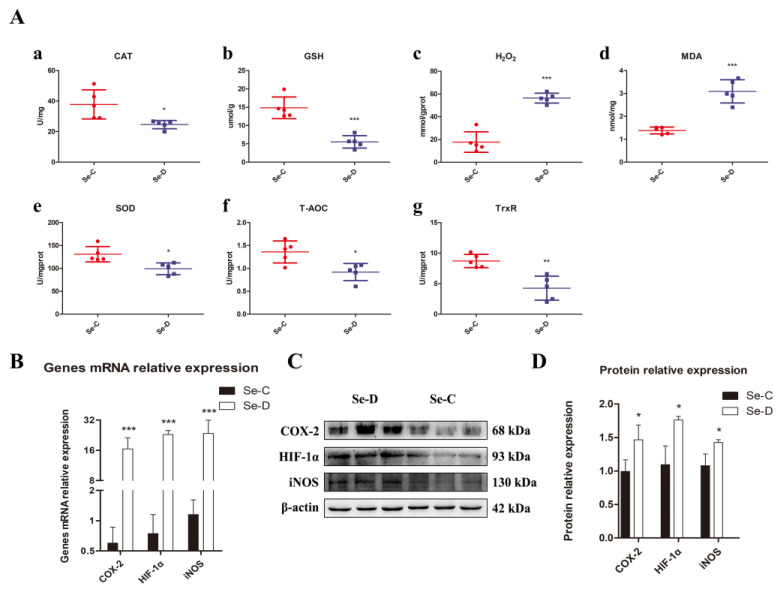
The antioxidant capacity of the lungs is assessed by the assay of (**A**) CAT activity (a), GSH concentration (b), H_2_O_2_ concentration (c), MDA concentration (d), SOD activity (e), T-AOC (f), and TrxR activity (g). Gene expression and protein levels of oxidative stress-related protein *COX-2*, *HIF-1α*, and *iNOS*, are examined by RT-qPCR (**B**) and WB (**C**,**D**). The mean ± SD is used to present the data above, *n* = 5 calves per group (except for WB *n* = 3). * *p* < 0.05, ** *p* < 0.01, *** *p* < 0.001.

The contents of GSH, T-AOC, MDA, and H_2_O_2_ and the activities of redox-related enzymes CAT, SOD, and TrxR were determined to investigate whether Se deficiency caused oxidative stress in the lungs of calves. It was found that in the lungs of the Se-D group, T-AOC and GSH levels were significantly lower than those in healthy calves. There was a significant decrease in SOD, CAT, and TrxR activities, indicating that the antioxidant capacity of their lungs was impaired. MDA and H_2_O_2_, by-products of oxidative stress, were distinctly elevated compared with the Se-C group (Figure 3A). To further understand the oxidative stress in the lung, gene and protein expression levels of important indicators related to antioxidants were investigated by RT-qPCR and WB, including HIF-1α, iNOS, and COX-2. At gene and protein levels, the Se-D group had significantly higher levels of iNOS, HIF-1α, and COX-2 compared with the Se-C group (Figure 3B–D). These results suggested that Se deficiency triggers a severe redox imbalance in the lungs of calves, leading to a significant decrease in antioxidant capacity.

### 3.4. Se Deficiency Activates the Caspase-8 and Caspase-9 Pathways to Trigger Apoptosis in Lung Cells

**Figure 4:**
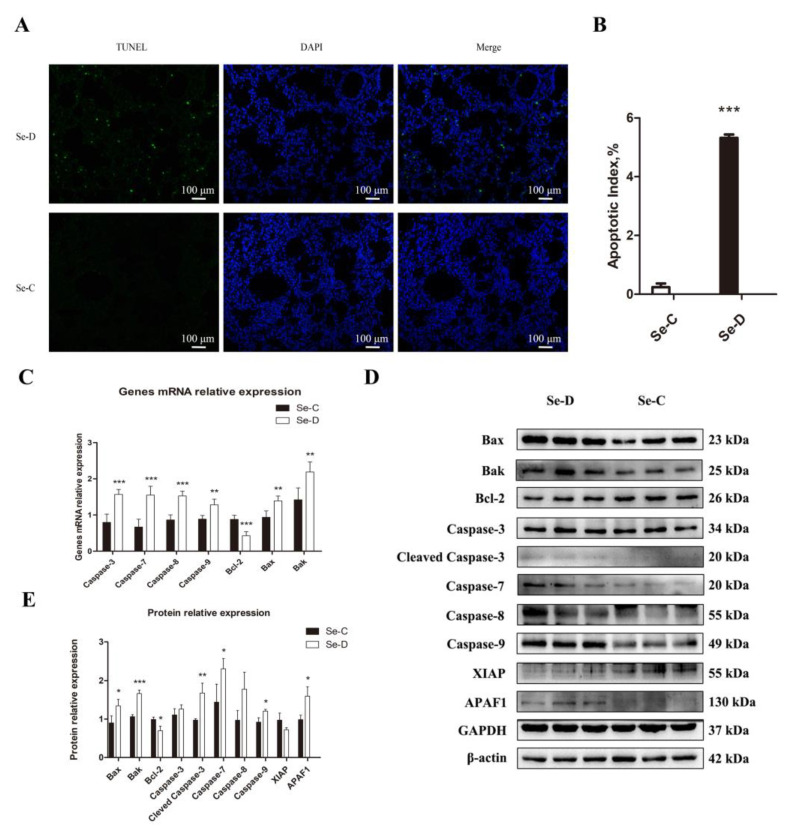
TUNEL staining (**A**) demonstrates the effect of Se deficiency on apoptosis in calf lung. (**B**) Apoptotic index of Se-C and Se-D groups. (**C**) mRNA expression of genes involved in apoptosis. (**D**,**E**) Expression of apoptosis-related proteins and their quantification. The mean ± SD is used to present the data above, *n* = 5 calves per group (except for WB *n* = 3). * *p* < 0.05, ** *p* < 0.01, *** *p* < 0.001.

The pathological damage and the oxidative stress in the lung prompted further studies on regulated cell death. An investigation of apoptosis caused by Se deficiency was conducted using TUNEL staining. The Se-C group had only 0.24% apoptotic cells, whereas the Se-D group had a higher apoptosis percentage of 5.32% (Figure 4A,B). In addition, the Se-D group had a higher mRNA expression level of Caspase-8, Caspase-9, Caspase-3, Caspase-7, Bax, and Bak compared with the Se-C group, suggesting more apoptosis in the Se-D group. Bcl-2, as an antiapoptotic gene, decreased significantly in the Se-D group (Figure 4C). Furthermore, 10 apoptosis-related proteins were detected by WB. As shown in Figure 4D,E, the Se-D group showed higher levels of cleaved Caspase-3, Caspase-7, Caspase-9, Bax, Bak, and APAF1 compared with the Se-C group. (Figure 4D,E). The results indicated that Se-deficient calves exhibit a higher proportion of apoptosis in their lungs.

### 3.5. NF-κB and MAPK Pathways Were Activated to Promote Lung Inflammation in Se Deficiency

**Figure 5:**
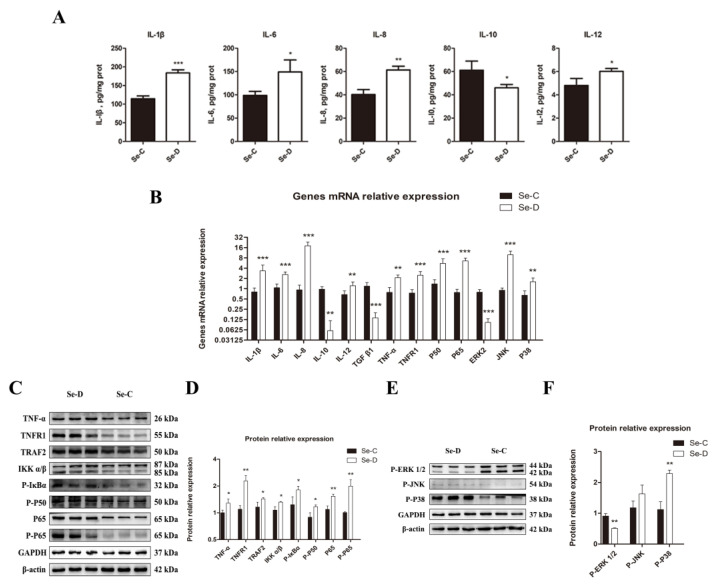
Inflammatory factor levels (**A**) in the lungs of calves were detected by ELISA. (**B**) Expression levels of cytokines, NF-κB, and MAPK pathway related genes. The levels of NF-κB (**C**,**D**) and MAPK (**E**,**F**) pathways related proteins are detected and their quantification. The mean ± SD is used to present the data above, *n* = 5 calves per group (except for WB *n =* 3). * *p* < 0.05, ** *p* < 0.01, *** *p* < 0.001.

Because cytokines are signaling molecules that can directly reflect inflammation in vivo, the present study used an ELISA to measure the concentrations of IL-1β, IL-6, IL-8, IL-10, and IL-12. The Se-D group had a significant increase in pro-inflammatory cytokine levels of IL-1β, IL-8, and IL-12. In contrast, the level of the anti-inflammatory cytokine IL-10 decreased markedly in the Se-D group. The lungs in the Se-D group had significantly higher levels of IL-6, a key cytokine involved in both pro- and anti-inflammatory processes. (Figure 5A). The Se-D group showed a significant decrease in TGF-β1 gene expression. The relative mRNA expression levels of cytokines were in general agreement with the results of the ELISA assay (Figure 5B). Key molecules involved in NF-κB and MAPK signaling pathways were detected by RT-qPCR and WB because the inflammation was strongly associated with both signaling pathways. The Se-D group showed higher gene levels of TNF-α, TNFR1, P50, and P65 compared with the Se-C group. TNFR1, TRAF2, TNF-α, IKKα/β, P-IκBα, P-P50, P65, and P-P65 protein levels showed an increase in the Se-D group (Figure 5C,D). In addition, the Se-D group had significantly higher expression levels of MAPK pathway-related genes JNK and P38, whereas ERK2 was significantly decreased. The Se-D group showed a decrease in P-ERK1/2 and an increase in P-P38 at the protein level. Similarly, the Se-D group also had elevated expression of P-JNK, but not significantly so (Figure 5E,F).

### 3.6. Necroptosis Levels Were Significantly Elevated in the Lungs of Se-Deficient Calves

**Figure 6:**
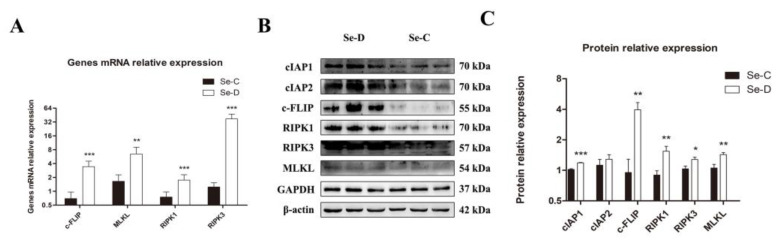
The mRNA (**A**) and their protein levels (**B**,**C**) of necroptosis-related genes are examined by RT-qPCR and WB, respectively. The mean ± SD is used to present the data above, *n =* 5 calves per group (except for WB *n =* 3). * *p* < 0.05, ** *p* < 0.01, *** *p* < 0.001.

As one of the types of regulated cell death, necroptosis was further investigated in Se deficiency at the molecular level. The mRNAs expression levels of four critical genes involved in necroptosis were determined by RT-qPCR. The study found that the mRNA levels of c-FLIP (FLICE inhibitory protein), MLKL, RIPK1, and RIPK3 were significantly higher in the lungs of Se-deficient calves (Figure 6A). Expression levels of 6 proteins associated with necroptosis were identified by WB. The Se-D group exhibited higher levels of cIAP1, c-FLIP, RIPK1, RIPK3, and MLKL. Compared with the Se-C group, the cIAP2 protein was slightly elevated in the Se-D group, but the difference did not reach statistical significance (Figure 6B,C).

## 4. Discussion

Se is an essential component as well as the active center of selenoprotein. The present study found that inadequate dietary Se led to a decrease in blood and lung Se levels in calves, and the effects of dietary Se on tissues were generally consistent with a previous study [20]. A decrease in Se is detrimental to the synthesis and activity of selenoproteins [22]. Changes in selenoprotein mRNA expression in the lungs differed slightly from the results in the hearts of selenium-deficient calves [20]. The synthesis priority and distribution differences in the organs of selenoproteins caused differences in expression levels during Se deficiency [23,24]. For the reason that the biological function of selenoproteins in vivo is largely related to antioxidants, Se deficiency always results in redox impairment and, eventually, lipid, DNA, and protein damage. The current study found that calves with Se deficiency had significantly lower Se concentrations in their lungs than healthy calves, which was accompanied by a decrease in the expression levels of 11 selenoproteins, including *GPX1*, *GPX4*, *SEL 15*, *SEL X*, *SEL W*, *SEL T*, *SEL P*, *SEL O*, *SEL M*, *DIO1*, and *DIO3*. *GPX* is a series of antioxidant enzymes involved in lipid peroxidation, detoxification, and other redox processes. *GPX1* is crucial for maintaining intracellular H_2_O_2_ balance because it reduces H_2_O_2_ to H_2_O [25]. *GPX4* homozygous knockout mice have been shown to be embryonic lethal because the membrane phospholipid hydroperoxides are reduced by this gene [26]. *SEL 15* is an ER-resident protein containing a thioredoxin-like fold with tissue specificity that is regulated by dietary Se [27]. Overexpression of *SEL M* in neuronal cells resists hydrogen peroxide-induced oxidative damage, and knockdown of *SEL M* results in decreased cell survival and strong apoptosis [28]. It has been shown that silencing of *SEL O* inhibits cartilage differentiation and proliferation and induces chondrocyte necrosis through apoptosis, which might be one of the major causes of endemic osteoarthropathy caused by Se deficiency [29]. *SEL P* is a secreted selenoprotein that is involved in the dynamic balance and distribution of Se in the whole body [30]. *SEL W* and *SEL T* are members of the RDX protein family and are thought to be thiol-based oxidoreductases, though their exact function is unknown [31]. As a zinc-containing selenoprotein, low *SEL X* expression reduces macrophage function, resulting in decreased natural immunity [32]. The *DIO* degrades T3, which playsf a key role in maintaining thyroid hormone activity and level. *DIO2* has been shown to be up-regulated in expression during acute and chronic inflammation [33], which is generally consistent with the results we detected. Remarkably, this study found that the expressions of 10 selenoprotein genes, including *GPX2*, *GPX3*, *SEL S*, *SEL N*, *SEL K*, *SEL I*, *SEL H*, *TXNRD1*, *TXNRD2*, and *TXNRD3*, were not significantly different. Most eukaryotes have two independent antioxidant systems: glutathione and thioredoxin, which are both closely related to selenoproteins. The thioredoxin system includes Trx, TrxRs, and NADPH. Trx is responsible for the reduction of disulfide bonds, while TrxRs are responsible for the reduction of oxidized Trx to make the reduction system sustainable [34]. In this experiment, a series of TrxRs proteins did not change significantly in mRNA and protein levels, but we found a significant decrease in TrxR activity in the next experiments, which may be due to the misincorporation of Cys into protein synthesis due to Sec deficiency, resulting in reduced antioxidant capacity [35,36]. The expression of *SEPHS2* was significantly elevated, which is generally in accordance with previous studies. For the reason that the *SEPHS2* catalyzes the synthesis of active Se donors, which is required for Sec, *SEPHS2* contributes to the abundance of selenoproteins [37]. Therefore, we speculate that the elevation of *SEPHS2* may be compensatory for the demand for other selenoproteins [38]. Overall, the differently expressed selenoprotein genes found in this experiment imply more research on redox function in Se-deficient bovine lungs is warranted.

The present study examined CAT, SOD, TrxR, reduced GSH, and T-AOC, given that Se deficiency may cause oxidative stress. Their decrease and the increase in H_2_O_2_ and MDA indicate oxidative stress in the lungs of Se-deficient calves. TrxR regulates inflammation and cell death through the MAPK signaling pathway [39]. H_2_O_2_ has a crucial role in the regulation of apoptosis, stress, and mitochondrial function [40]. Too much H_2_O_2_ causes lipid peroxidation, leading to the production of a variety of reactive aldehydes, including MDA. These aldehydes with ultra-long metabolic cycles enhance the initial free radical events, subsequently causing damage to the organism [41]. It has been suggested that the upregulation of MDA may correlate with the *GPX4* downregulation because *GPX4* is mainly responsible for the breakdown of lipid peroxides [26]. As the name implies, the main function of *iNOS* is to synthesize NO. Increased NO levels or decreased SOD activity results in the formation of large amounts of H_2_O_2_ that injure organs [42]. According to our findings, *iNOS* was dramatically upregulated in the Se-D group. Apparently, this becomes one of the important causes of Se deficiency-induced lung injury. *HIF-1α* is crucial for energy metabolism because it regulates glucose metabolism and oxygen homeostasis [43]. Increased endogenous NO has been shown to directly enhance *HIF-1α* stability and activity [44]. This finding coincides with our observation that elevated levels of *iNOS* are accompanied by significantly elevated *HIF-1α*. Although *HIF-1α* facilitates the mitigation of damage caused by hypoxia, sustained *HIF-1α* upregulation facilitates inflammation and oxidative stress [45,46]. The relationship between oxidative stress and inflammation in Se deficiency will be discussed in the following paragraph. *COX-2* is abundantly expressed in inflamed tissues [47] and has been extensively studied as a target for non-steroidal anti-inflammatory drugs. Elevated *COX-2* levels were observed in the lungs of Se-deficient calves. This is consistent with pathological findings in which the lungs are accompanied by a large infiltration of inflammatory cells. It has been shown that *COX-2* gene transcription is associated with MAPK pathway activation [48]. The relationship between *COX-2* and inflammation will be described in the next section.

The lung pathological findings in the Se-D group suggested an inflammatory process in the lungs. Therefore, IL-1β, IL-6, IL-8, IL-10, and IL-12 were detected by ELISA in the Se-C and Se-D groups. The Se-D group had significantly higher levels of IL-12, IL-8, IL-6, and IL-1β, reflecting the inflammation in the lungs. Meanwhile, the anti-inflammatory cytokine IL-10 was markedly reduced in the Se-deficient bovine lungs. The WB assay also revealed that the important pro-inflammatory cytokine *TNF-α* was significantly upregulated. This study measured the protein levels in the NF-κB pathway that have been shown to be activated by *TNF-α.* Our research indicated that *P-P65*, *P65*, *TNFR1*, *TRAF2*, *IKKα/β*, and *P-IκBα* expression levels were significantly higher in the Se-D group, indicating that the NF-κB pathway was activated in the lungs of Se-deficient calves. *TNF-α* is not the only activator of the NF-κB pathway; IL-1β, ROS, and H_2_O_2_ also activate this pathway to control the transcription of various genes, including *iNOS* and *COX-2*, to regulate inflammation. In this study, elevated *P38* and *JNK* suggested that the MAPK pathway is activated in the presence of Se deficiency. Interestingly, the Se-D group had a significant decrease in *ERK*, which may be associated with impaired cell proliferation capacity [49]. The anti-inflammatory function of dexamethasone includes the reduction of the mRNA stability of *COX-2* by blocking the *P38* pathway [48]. Based on previous studies and our results, oxidative stress induced by Se deficiency caused tissue inflammation via NF-κB and MAPK activation.

Regulated cell death often occurs when cells are subjected to internal or external stresses such as infection, oxidative stress, etc. According to current research, we investigated the apoptosis and necroptosis caused by Se deficiency. According to TUNEL staining, the Se-D group showed more severe apoptosis than the Se-C group. The upregulation of *Caspase-3*, *Caspase-7*, *Caspase-8*, *Caspase-9*, *Bax*, and *Bak* and the downregulation of *Bcl-2* were found in the Se-D group, indicating that Se deficiency induced apoptosis in lung cells through the *Caspase-8* and *Caspase-9* pathways [50,51]. H_2_O_2_ can rapidly activate *ERK1/2*, *JNK*, and *p38* to induce apoptosis [52]. In contrast to apoptosis, necroptosis is dependent on *RIPK3* [53]. In our study, the Se-D group showed higher levels of *cIAP1*, *c-FLIP*, *RIPK1*, *RIPK3*, and *MLKL* mRNA expression than the control, suggesting that necroptosis in lung cells was activated in Se deficiency. *FLIP*, an apoptosis-regulating protein in the Fas death pathway, resembles *Caspase-8* in structure but lacks its function. The level of *c-FLIP* has an impact on its function [51]. *Caspase-8* dimer formation was promoted at low *c-FLIP* levels, inducing apoptosis, whereas heterodimer formation with *Caspase-8* inhibited both apoptosis and necroptosis at high levels of *c-FLIP*. The present study found that *c-FLIP* was significantly upregulated at both the mRNA and protein levels in the Se-D group, but it failed to inhibit apoptosis and necroptosis. Up-regulated *c-FLIP* may be associated with the protective function of an activated NF-κB pathway in cells [54].

In conclusion, the present study found that Se deficiency caused changes in selenoprotein expression, inducing oxidative stress, which in turn triggered inflammation, apoptosis, and necroptosis, eventually causing tissue injury in weaned calves’ lungs.

## Data Availability

The original contributions presented in the study are included in the article/supplementary material. Further inquiries can be directed to the corresponding authors.

## Supplementary Materials

The following supporting information can be downloaded at: https://www.mdpi.com/article/10.3390/antiox12040796/s1, Table S1: The antibodies used in the present study.

## References

[1] Yang C., Yao H., Wu Y., Sun G., Yang W., Li Z. (2021). Status and risks of selenium deficiency in a traditional selenium-deficient area in Northeast China. Sci. Total Environ..

[2] Mehdi Y., Dufrasne I. (2016). Selenium in Cattle: A Review. Molecules.

[3] Labunskyy V.M., Hatfield D.L., Gladyshev V.N. (2014). Selenoproteins: Molecular pathways and physiological roles. Physiol. Rev..

[4] Markley R.L., Restori K.H., Katkere B., Sumner S.E., Nicol M.J., Tyryshkina A., Nettleford S.K., Williamson D.R., Place D.E., Dewan K.K. (2021). Macrophage selenoproteins restrict intracellular replication of francisella tularensis and are essential for host immunity. Front Immunol..

[5] Gozzi-Silva S.C., Teixeira F.M.E., Duarte A.J.D.S., Sato M.N., Oliveira L.M. (2021). Immunomodulatory role of nutrients: How can pulmonary dysfunctions improve?. Front. Nutr..

[6] Garry M.R., Kavanagh T.J., Faustman E.M., Sidhu J.S., Liao R., Ware C. (2008). Sensitivity of mouse lung fibroblasts heterozygous for GPx4 to oxidative stress. Free Radic. Biol. Med..

[7] Amaral E.P., Foreman T.W., Namasivayam S., Hilligan K.L., Kauffman K.D., Barbosa Bomfim C.C. (2022). GPX4 regulates cellular necrosis and host resistance in Mycobacterium tuberculosis infection. J. Exp. Med..

[8] Tindell R., Wall S.B., Li Q., Li R., Dunigan K., Wood R. (2018). Selenium supplementation of lung epithelial cells enhances nuclear factor E2-related factor 2 (Nrf2) activation following thioredoxin reductase inhibition. Redox Biol..

[9] Tsuji P., Carlson B., Anderson C., Seifried H., Hatfield D., Howard M. (2015). Dietary selenium levels affect selenoprotein expression and support the interferon-γ and IL-6 immune response pathways in mice. Nutrients.

[10] Moghadaszadeh B., Rider B.E., Lawlor M.W., Childers M.K., Grange R.W., Gupta K. (2013). Selenoprotein N deficiency in mice is associated with abnormal lung development. FASEB J..

[11] Holmgren A. (2000). Antioxidant function of thioredoxin and glutaredoxin systems. Antioxid. Redox Signal..

[12] Zhang Q., Lenardo M.J., Baltimore D. (2017). 30 Years of NF-kappaB: A blossoming of relevance to human pathobiology. Cell.

[13] Morgan M.J., Liu Z.G. (2011). Crosstalk of reactive oxygen species and NF-kappaB signaling. Cell Res..

[14] Sun Y., Liu W.Z., Liu T., Feng X., Yang N., Zhou H.F. (2015). Signaling pathway of MAPK/ERK in cell proliferation, differentiation, migration, senescence and apoptosis. J. Recept. Signal Transduct. Res..

[15] Pagliari L.J., Pinkoski M.J., Green D.R. (2003). Apotosis signaling: A means to an end. Handb. Cell Signal..

[16] Dondelinger Y., Hulpiau P., Saeys Y., Bertrand M.J., Vandenabeele P. (2016). An evolutionary perspective on the necroptotic pathway. Trends Cell Biol..

[17] Darlow B.A., Winterbourn C.C., Inder T.E., Graham P.J., Harding J.E., Weston P.J., Sluis K.B. (2000). The effect of selenium supplementation on outcome in very low birth weight infants: A randomized controlled trial. J. Pediatr..

[18] Darlow B.A., Austin N.C. (2003). Selenium supplementation to prevent short-term morbidity in preterm neonates. Cochrane Database Syst. Rev..

[19] Sherlock L.G., McCarthy W.C., Grayck M.R., Solar M., Hernandez A., Zheng L., Delaney C., Tipple T.E., Wright C.J., Nozik E.S. (2022). Neonatal selenium deficiency decreases selenoproteins in the lung and impairs pulmonary alveolar development. Antioxidants.

[20] Lei L., Mu J., Zheng Y., Liu Y. (2023). Selenium deficiency-induced oxidative stress causes myocardial injury in calves by activating inflammation, apoptosis, and necroptosis. Antioxidants.

[21] Hawker F.H., Ward H.E., Stewart P.M. (1993). Selenium deficiency augments the pulmonary toxic effects of oxygen exposure in the rat. Eur. Respir. J..

[22] Fordyce F.M. (2013). Selenium deficiency and toxicity in the environment. Essentials of Medical Geology.

[23] Hill K.E., Lyons P.R., Burk R.F. (1992). Differential regulation of rat liver selenoprotein mRNAs in selenium deficiency. Biochem. Biophys. Res. Commun..

[24] Wingler K., Böcher M., Flohé L., Kollmus H., Brigelius-Flohé R. (1999). mRNA stability and selenocysteine insertion sequence efficiency rank gastrointestinal glutathione peroxidase high in the hierarchy of selenoproteins. Eur. J. Biochem..

[25] Lubos E., Loscalzo J., Handy D.E. (2012). Glutathione peroxidase-1 in health and disease: From molecular mechanisms to therapeutic opportunities. Antioxidants Redox Signal..

[26] Yant L.J., Ran Q., Rao L., Remmen H.V., Shibatani T., Belter J.G. (2003). The selenoprotein GPX4 is essential for mouse development and protects from radiation and oxidative damage insults. Free Radic. Biol. Med..

[27] Korotkov K.V., Kumaraswamy E., Zhou Y., Hatfield D.L., Gladyshev V.N. (2001). Association between the 15-kDa selenoprotein and UDP-glucose:glycoprotein glucosyltransferase in the endoplasmic reticulum of mammalian cells. J. Biol. Chem..

[28] Reeves M.A., Bellinger F.P., Berry M.J. (2010). The Neuroprotective Functions of Selenoprotein M and its Role in Cytosolic Cal. cium Regulation. Antioxid. Redox Signal..

[29] Yan J., Fei Y., Han Y., Lu S. (2016). Selenoprotein O deficiencies suppress chondrogenic differentiation of ATDC5 cells. Cell Biol. Int..

[30] Burk R.F., Hill K.E. (2009). Selenoprotein P-expression, functions, and roles in mammals. Biochim. Biophys. Acta.

[31] Dikiy A., Novoselov S.V., Fomenko D.E., Sengupta A., Carlson B.A., Cerny R.L. (2007). SelT, SelW, SelH, and Rdx12: Genomics and molecular insights into the functions of selenoproteins of a novel thioredoxin-like family. Biochemistry.

[32] Lee B.C., Peterfi Z., Hoffmann F.W., Moore R.E., Kaya A., Avanesov A. (2013). MsrB1 and MICALs regulate actin assembly and macrophage function via reversible stereoselective methionine oxidation. Mol. Cell.

[33] Dentice M., Marsili A., Ambrosio R., Guardiola O., Sibilio A., Paik J.H. (2010). The FoxO3/type 2 deiodinase pathway is required for normal mouse myogenesis and muscle regeneration. J. Clin. Investig..

[34] Sun Q.A., Kirnarsky L., Sherman S., Gladyshev V.N. (2001). Selenoprotein oxidoreductase with specificity for thioredoxin and glutathione systems. Proc. Natl. Acad. Sci. USA.

[35] Gromer S., Johansson L., Bauer H., Arscott L.D., Rauch S., Ballou D.P. (2003). Active sites of thioredoxin reductases: Why selenoproteins?. Proc. Natl. Acad. Sci. USA.

[36] Xu X.M., Turanov A.A., Carlson B.A., Yoo M.H., Everley R.A., Nandakumar R. (2015). Targeted insertion of cysteine by decoding UGA codons with mammalian selenocysteine machinery. Proc. Natl. Acad. Sci. USA.

[37] Xu X.M., Carlson B.A., Irons R., Mix H., Zhong N., Gladyshev V.N. (2007). Selenophosphate synthetase 2 is essential for selenoprotein biosynthesis. Biochem. J..

[38] Li S., Zhao Q., Zhang K., Sun W., Jia X., Yang Y. (2020). Se deficiency induces renal pathological changes by regulating selenoprotein expression, disrupting redox balance, and activating inflammation. Metallomics.

[39] Chang L., Karin M. (2001). Mammalian MAP kinase signalling cascades. Nature.

[40] Autreaux B.D., Toledano M.B. (2007). ROS as signalling molecules: Mechanisms that generate specificity in ROS homeostasis. Nat. Rev. Mol. Cell Biol..

[41] Esterbauer H., Schaur R.J., Zollner H. (1991). Chemistry and biochemistry of 4-hydroxynonenal, malonaldehyde and related aldehydes. Free Radic. Biol. Med..

[42] Bonfoco E., Krainc D., Ankarcrona M., Nicotera P., Lipton S.A. (1995). Apoptosis and necrosis: Two distinct events induced, respectively, by mild and intense insults with N-methyl-D-aspartate or nitric oxide/superoxide in cortical cell cultures. Proc. Natl. Acad. Sci. USA.

[43] Semenza G.L. (2012). Hypoxia-inducible factors in physiology and medicine. Cell.

[44] Movafagh S., Crook S., Vo K. (2015). Regulation of hypoxia-inducible factor-1a by reactive oxygen species: New developments in an old debate. J. Cell Biochem..

[45] Packer M. (2020). Mutual Antagonism of Hypoxia-Inducible Factor Isoforms in Cardiac, Vascular, and Renal Disorders. JACC Basic Transl. Sci..

[46] Luo L., Luo G., Fang Q., Sun Z. (2014). Stable expression of hypoxia-inducible factor-1alpha in human renal proximal tubular epithelial cells promotes epithelial to mesenchymal transition. Transpl. Proc..

[47] Brenhouse H.C., Andersen S.L. (2011). Nonsteroidal anti-inflammatory treatment prevents delayed effects of early life stress in rats. Biol. Psychiatry.

[48] Cok S.J., Morrison A.R. (2001). The 3′-untranslated region of murine cyclooxygenase-2 contains multiple regulatory elements that alter message stability and translational efficiency. J. Biol. Chem..

[49] Aoki K., Kumagai Y., Sakurai A., Komatsu N., Fujita Y., Shionyu C. (2013). Stochastic ERK activation induced by noise and cell-to-cell propagation regulates cell density-dependent proliferation. Mol. Cell.

[50] Marchi S., Giorgi C., Suski J.M., Agnoletto C., Bononi A., Bonora M. (2012). Mitochondria-ros crosstalk in the control of cell death and aging. J. Signal Transduct..

[51] Tummers B., Green D.R. (2022). The evolution of regulated cell death pathways in animals and their evasion by pathogens. Physiol. Rev..

[52] Chen L., Liu L., Yin J., Luo Y., Huang S. (2009). Hydrogen peroxide-induced neuronal apoptosis is associated with inhibition of protein phosphatase 2A and 5, leading to activation of MAPK pathway. Int. J. Biochem. Cell Biol..

[53] Shubina M., Tummers B., Boyd D.F., Zhang T., Yin C., Gautam A. (2020). Necroptosis restricts influenza A virus as a stand-alone cell death mechanism. J. Exp. Med..

[54] Kreuz S., Siegmund D., Scheurich P., Wajant H. (2001). NF-kappaB inducers upregulate cFLIP, a cycloheximide-sensitive inhibitor of death receptor signaling. Mol. Cell. Biol..

