# Supplementary material for: Oxidative Stress Induced by Selenium Deficiency Contributes to Inflammation, Apoptosis and Necroptosis in the Lungs of Calves
Source: Antioxidants (Basel). 2023 Mar 24;12(4):796. doi: 10.3390/antiox12040796 (PMC10135166; doi:10.3390/antiox12040796)
Supplement: Supplementary file 1 [file antioxidants-12-00796-s001.zip › antioxidants-2273272-supplementary.pdf]

Oxidative stress induced by selenium deficiency contributes to inflammation, apoptosis and necroptosis in the lungs of calves

Jing Mu

Table S1 The antibodies used in the present study

Antibody	Brand	Cat.	Antibody	Brand	Cat.
GPX1	Wanleibio	WL02497a	P65	Wanleibio	WL01980
COX-2	Wanleibio	WL01750	P-P65	Wanleibio	WL02169
iNOS	Wanleibio	WL0992a	P-ERK1/2	Wanleibio	WLP1512
Bax	Wanleibio	WL01637	P-JNK	Wanleibio	WL01813
Bak	Wanleibio	WL0129a	P-P38	Wanleibio	WLP1576
Bcl-2	Wanleibio	WL01556	cIAP1	Wanleibio	WL03666
Caspase-3	Wanleibio	WL02117	cIAP2	Wanleibio	WL01254
Caspase-7	Wanleibio	WL02360	c-FILP	Wanleibio	WL02485
Caspase-8	Wanleibio	WL03426	HIF-1α	Bioss	BS-0737R
Caspase-9	Wanleibio	WL03421	RIPK1	Bioss	BS-5805R
XIAP	Wanleibio	WL03561	RIPK3	Bioss	BS-3551R
APAF1	Wanleibio	WL04536	β-actin	ABclonal	AC006
TNF-α	Wanleibio	WL01581	GAPDH	ABclonal	AC001
TNFR1	Wanleibio	WL01414	GPX4	Abcam	ab41787
TRAF2	Wanleibio	WL02846	TXNRD3	Proteintech	19517-1-AP
IKK α/β	Wanleibio	WL01900	MLKL	Proteintech	21066-1-AP
P-IκBα	Wanleibio	WL02495	Cleaved Caspase-3	CST	9661T
P-P50	Wanleibio	WL01866	Goat Anti-Rabbit IgG (H+L)	ABclonal	AS014

Note: Wanleibio, Shenyang, China; Bioss, Beijing, China; Proteintech, Rosemont, IL, USA; ABclonal, Wuhan, China; Abcam, Cambridge, UK; CST, Beverly, MA, USA.
